# Preparation of Low-Molecular-Weight Polyacrylamide as the Delayed Crosslinking Plugging Agent for Drilling Fluid

**DOI:** 10.3390/gels10020112

**Published:** 2024-02-01

**Authors:** Quanyang Wang, Chenghua Zhou, Honghu Zhang, Xue Zhang, Xinxin Wen, Jiexin Bai, Hui Mao

**Affiliations:** 1 Drilling Engineering Research Institute, Sinopec Xinan Oilfield Service Corporation, Deyang 618000, China; wangqy.osxn@sinopec.com (Q.W.); zhouch.osxn@sinopec.com (C.Z.); zhanghh2312.osxn@sinopec.com (H.Z.); zhangxue.osxn@sinopec.com (X.Z.); 2 College of Energy Resources, Chengdu University of Technology, Chengdu 610059, China; 201704010427@stu.cdut.edu.cn (X.W.); baijiexin@stu.cdut.edu.cn (J.B.)

**Keywords:** delayed crosslinking, low viscosity, plugging material, polymer gel, well leakage

## Abstract

Deep wells and ultra-deep wells often encounter cracks, karst caves, and other developed strata, which can lead to leakage during drilling. Conventional bridge slurry plugging technology is prone to leaking due to the poor plugging effect of the plugging agent. The gel plugging agent possesses characteristics of flexible plugging and adaptive matching of formation leakage channels. It can fill cracks or caves and enhance the pressure-bearing capacity of the formation. A controllable crosslinking plugging agent based on low-molecular-weight polyacrylamide was studied. Polyacrylamide with different molecular weights is synthesized from acrylamide and an initiator. A crosslinking time-controllable polymer is synthesized from low-molecular-weight polyacrylamide by adding crosslinking agent and retarder. The low-molecular-weight polyacrylamide plugging agent has low viscosity before gelation and good fluidity in the wellbore. After being configured on the ground, it is transported by pipeline and sent underground to reach the thickening condition. The gel solution rapidly solidifies, and its strength improves after high-temperature crosslinking. The synthesis conditions of the polymer were as follows: a monomer concentration of 9%, initiator 3.5%, synthesis temperature of 65 °C, and hydrogen peroxide initiator. The optimal formula of the gel plugging agent is as follows: a polymer concentration of 6%, a crosslinking agent concentration of 1%, and a retarder concentration of 8%. The generated polymer molecular structure contains amide groups. This crosslinking time-controllable plugging agent based on low-molecular-weight polyacrylamide has stable rheology, and its temperature resistance can reach 150 °C. At 150 °C, the gelation time can be controlled by adjusting the concentration of retarder, and the longest can reach 4 h. The plugging efficiency of the gel plugging agent is more than 95%. With the increase in seam width, the pressure of the gel plugging agent gradually decreases.

## 1. Introduction

With the deepening exploration and development of oil and gas resources, the cost of handling well leaks has significantly increased [1,2,3]. Formation leaks not only affect drilling efficiency but also lead to unstable wellbore collapse and, in severe cases, the blockage of flow channels, resulting in reduced oil well production [4,5,6,7]. Well leaks, especially malignant leaks, limit the efficient exploration, development, and utilization of oil and gas in our country [8]. Currently, both domestic and international approaches to addressing malignant leaks mainly include bridge plug technology, composite plug technology, high-loss water plug technology, cement slurry plug technology, and gel plug technology, etc. [9,10,11,12,13]. However, these technologies, such as bridge plug, composite plug, and high-loss water plug, face a common challenge: they are difficult to retain in channels of malignant leaks and have poor sealing effects in formations with fracture development and fragmentation [14,15,16]. When using traditional bridge plug agents [17,18,19], their pressure-bearing capacity is relatively low. When using cement slurry plug agents [20], it is difficult for them to stay in leakage channels, and they are easily diluted and washed away by formation water. As a result, the solidification effect becomes worse, and it is difficult to form a tight plugging layer with sufficient strength around the wellbore, leading to decreased sealing effectiveness [21,22].

The gel plugging material has been maturely applied in the field, and its advantages are obvious. First, the scope of application is wide: whether it is pore-type leakage or fracture-type leakage, gel plugging materials can be applied. Because of the excellent deformation ability of the gel, regardless of the size and geometry of the leakage channel, it can enter through deformation and extrusion to form a dense plugging layer to prevent the pressure propagation and induced expansion of the fracture. Second, it is well compounded with bridge plugging materials. After adding particle plugging materials to the gel, it will interact with the groups on the gel polymer and greatly enhance the strength of the gel, and the plugging effect will be better. Third, the gel has strong viscous resistance and anti-shear dilution ability. After entering the formation, its spatial network structure will make the gel adsorb on the surface of the formation and will not be diluted by the water phase. Finally, the gel plugging material has good degradation ability, which can be removed by biodegradation, thermal degradation, acid degradation, and other methods, which is effective in protecting the reservoir [23,24].

One of the main problems of gel plugging agent is gelation time. When the formation is shallow, the problem of gelation time is rarely considered. Once the drilling operation is deep, if the gel plugging agent does not have enough retarding time, it will crosslink in the wellbore and then block the wellbore. Generally, the preparation of crosslinked polymer gel plugging agent uses fresh water, and the polymer and crosslinking agent are prepared separately. If the leakage is particularly serious, fiber, walnut shell, fruit shell, and other bridging plugging materials can be considered. There are two ways of gel crosslinking, downhole crosslinking and pre-crosslinking. The so-called downhole crosslinking involves pumping the plugging polymer material into the well and then using a small displacement jet pump to pump the diluted crosslinking agent at the riser to mix it with the polymer. When it flows through the formation, it will be subjected to the dual effects of formation shear and formation temperature and then crosslinked to form a high-strength gel. Pre-crosslinking involves compounding the crosslinking agent and the polymer on the ground, designing the gelation time, and then pumping it into the well after the mixing is completed. After reaching the leakage layer, the crosslinking is just completed to form a gel. Generally, pre-crosslinking is used when the well leakage is serious. The crosslinked gel is rubber-like, has good elasticity and viscosity, and can stay in the formation and form a plugging layer. In general, this gel can be degraded over time or degraded by acid dissolution [25]. After the gel enters the specified formation, it enters the formation fracture or hole through extrusion deformation, and the excellent shear resistance can ensure that the gel is not diluted by shear. If it carries fiber and other particle plugging materials, it can also play a role in bridging, while the gel enters small cracks. Because the gel has strong viscosity, it is easy for the gel to stay in the formation and form a tight plugging layer to prevent pressure from propagating in the cracks [26], so as to achieve the purpose of plugging the leakage.

In order to make up for the deficiency in the delayed crosslinking performance of gel plugging materials, in this paper, polyacrylamide and other monomers were used as raw materials to synthesize the main agent of a gel plugging agent, and low-molecular-weight polyacrylamide prepared with hydrogen peroxide as the initiator was used as a thickener to react with different concentrations of crosslinking agent alkaline chromium acetate and retarder sodium lactate to prepare a crosslinking time-controllable plugging agent based on low-molecular-weight polyacrylamide. 

The plugging agent has a low molecular weight, low viscosity, and good fluidity in the wellbore before gelation, so it can be transported to the downhole through a pipeline after configuration on the ground to achieve thickening conditions, effectively solving the problem that the polymer crosslinking time is not easy to control. The temperature resistance reaches 150 °C. Under the condition of 150 °C, the crosslinking time can be controlled by adjusting the concentration of retarder, the longest can reach 4 h, the rheological property is stable, and the upper limit of salt resistance is 15,000 mg/L. The plugging efficiency of the gel plugging agent is more than 95%.

## 2. Results and Discussion

### 2.1. Optimization of Low-Molecular-Weight Polyacrylamide

#### 2.1.1. Optimization of Initiator

The initiators used were hydrogen peroxide and ammonium persulfate. By varying the type and concentration of the initiators, the flowability of the gel solution formed by polymer configuration was observed, and viscoelasticity testing was conducted to measure its elastic modulus. The flowability descriptions of different initiators and the results of the elastic modulus are shown in Table 1.

From Table 1, it can be observed that the flowability and strength of the time-controlled crosslinkable plugging agent prepared from low-molecular-weight polyacrylamide using different initiators, hydrogen peroxide and ammonium persulfate, vary. The time-controlled crosslinkable plugging agent prepared using hydrogen peroxide exhibits good flowability before crosslinking, which is more advantageous for pumping during on-site construction processes. Furthermore, in terms of the strength after high-temperature crosslinking, the time-controlled crosslinkable plugging agent prepared using hydrogen peroxide and low-molecular-weight polyacrylamide demonstrates higher strength than the agent prepared using ammonium persulfate and low-relative-molecular-weight polyacrylamide. Therefore, hydrogen peroxide is preferred as the initiator for synthesizing low-molecular-weight polyacrylamide.

As the initiator concentration increases, the number of free radicals increases, but there are not enough monomers available to react with the free radicals. This leads to chain transfer reactions between monomer radicals and polymer radicals towards the initiator, ultimately causing the loss of reactivity and resulting in shorter polymer chains formed. As a result, the gel strength decreases [27].

#### 2.1.2. Optimization of Monomer Concentration

During the synthesis of the polymer, the monomer concentration gradually increased from 8% to 12% in sequential increments. After the polymer synthesis, viscoelasticity testing was conducted. 

**Figure 1:**
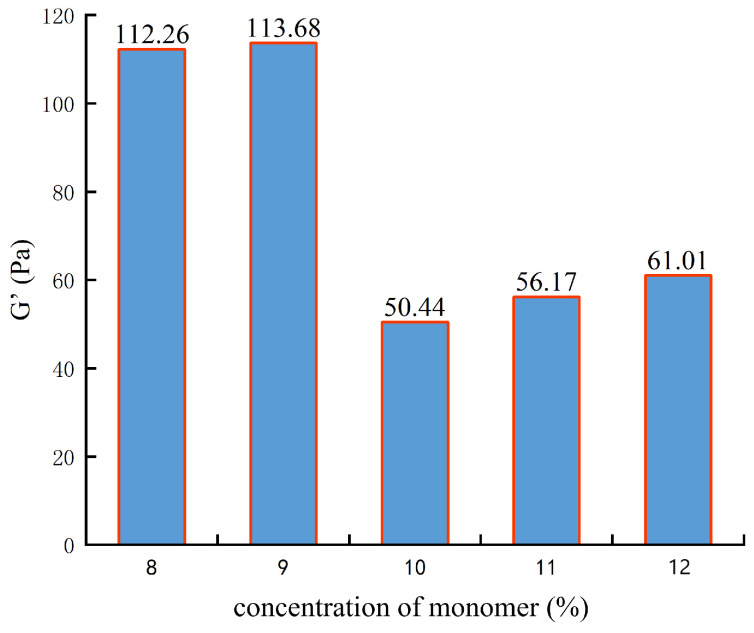
The experimental results of monomer concentration optimization.

It can be seen from Figure 1 that the elastic modulus was 112.26 Pa when the monomer concentration was 8%. When the monomer concentration increased to 9%, the elastic modulus of the gel basically did not change. When the monomer concentration continued to increase, the elastic modulus decreased sharply to 50 Pa, and then the elastic modulus increased slightly. When the monomer concentration is 9%, the elastic modulus is the largest. As the monomer concentration increases, the polymerization reaction rate accelerates, resulting in longer polymer chains and increased gel strength. However, when the monomer concentration exceeds a certain threshold, the polymerization reaction rate becomes excessively fast, leading to explosive polymerization. This ultimately results in the synthesis of many low-molecular-weight polymers, causing a decrease in the strength of the crosslinked gel. Therefore, the optimal monomer concentration determined is 9%.

#### 2.1.3. Optimization of Initiator Concentration

**Figure 2:**
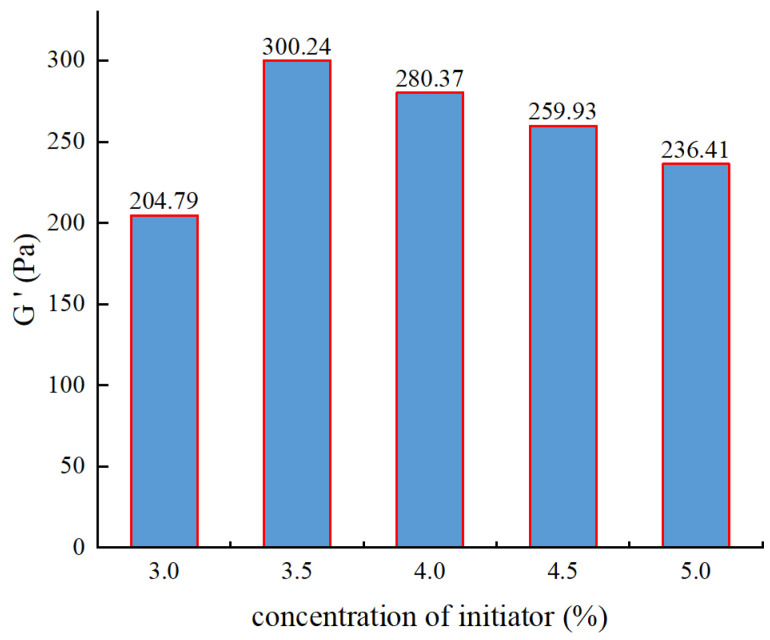
The experimental results of initiator concentration optimization.

During the polymer synthesis, the concentration of the H_2_O_2_ initiator was designed as 3%, 3.5%, 4%, 4.5%, and 5%. After the polymer synthesis, viscoelasticity testing was conducted as shown in Figure 2. 

From Figure 2, it can be observed that as the initiator concentration increases, the elastic modulus of the gel initially increases and then decreases. At an initiator concentration of 3.5%, the gel exhibits the maximum strength of 243.2 Pa. When the initiator concentration is low, fewer free radicals are generated from the decomposition of the initiator, resulting in incomplete monomer reactions. As the initiator concentration increases, the number of free radicals increases, but there are not enough monomers available to react with the free radicals. This leads to chain transfer reactions between monomer radicals and polymer radicals towards the initiator, ultimately causing the loss of reactivity and resulting in shorter polymer chains formed. As a result, the gel strength decreases. Therefore, the optimal initiator concentration determined is 3.5%.

#### 2.1.4. Optimization of Synthesis Temperature

**Figure 3:**
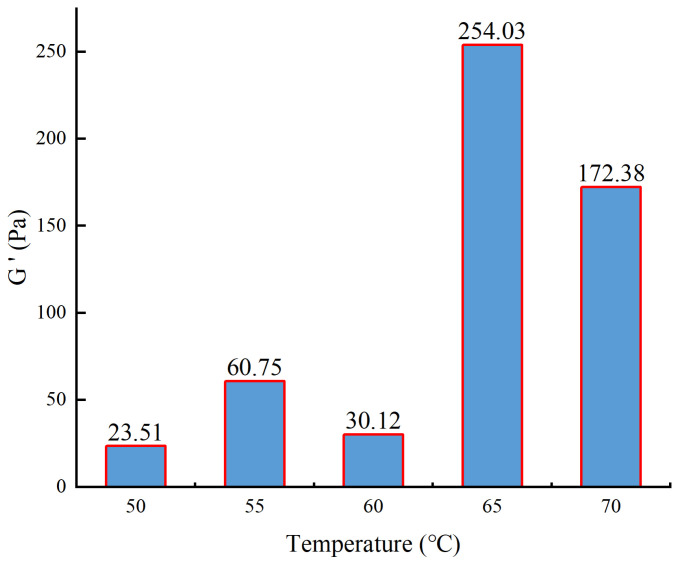
The experimental results of synthesis temperature.

The initiator needs to reach a certain temperature to crack to form free radicals. The optimization of the synthesis temperature was conducted as follows: the monomer concentration was 9%, the initiator concentration was 3.5%, and the synthesis temperature was changed to 50, 55, 60, 65, and 70 °C. The gel solution prepared with the polymer was thickened by a high-temperature and high-pressure thickener, and its elastic modulus was tested using a HAAKE rheometer. The experimental temperature was 30 °C, the experimental stress was 10 Pa, and the experimental frequency was 1 Hz. The results are shown in Figure 3. 

From Figure 3, it can be observed that as the synthesis temperature increases, the elastic modulus of the gel initially increases and then decreases. At a synthesis temperature of 65 °C, the gel exhibits the maximum strength. When the temperature is relatively low, the rate of initiator decomposition is slow, resulting in a lower number of generated free radicals. Consequently, there are insufficient free radicals available to react with the monomers, leading to incomplete monomer conversion even at the end of the reaction. As a result, the polymer chains do not grow significantly, resulting in a lower molecular weight and, consequently, reduced gel strength. As the temperature increases, more free radicals participate in the reaction, leading to longer polymer chains and increased gel strength. However, when the temperature exceeds 65 °C, the rate of initiator decomposition becomes excessively fast, leading to an excessive number of free radicals participating in the reaction. This can result in explosive polymerization, chain transfer, and chain termination reactions, causing a decrease in the molecular weight and a subsequent reduction in gel strength. Therefore, the optimal synthesis temperature determined is 65 °C.

### 2.2. Characterization of Low-Molecular-Weight Polyacrylamide

#### 2.2.1. Characterization of FT-IR

**Figure 4:**
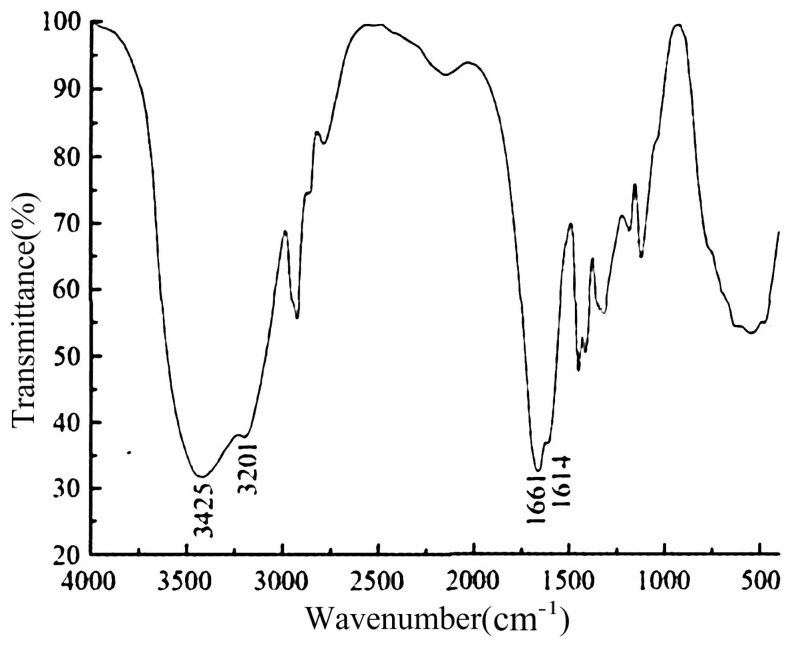
FT-IR image of polymer.

The results of infrared spectroscopy are shown in Figure 4. From Figure 4, it can be observed that the spectrum exhibits characteristic absorption peaks associated with the synthesized polyacrylamide. Specifically, at the wavenumber of 3425 cm^−1^, a peak corresponding to the free amide group (-NH_2_) is observed. At 3201 cm^−1^, a peak indicative of the bonded amide group (-NH_2_) is present. The wavenumber 1614 cm^−1^ corresponds to the bending vibration of the amide group (N-H), while the peak at 1661 cm^−1^ represents the characteristic absorption of the amide group (-C=O), specifically corresponding to the stretching vibration of the carbonyl group (C=O). These findings provide evidence of the successful synthesis of polyacrylamide, as the observed absorption peaks align with the expected spectral features of the amide functional groups present in the polymer.

#### 2.2.2. Gel Permeation Chromatography

**Figure 5:**
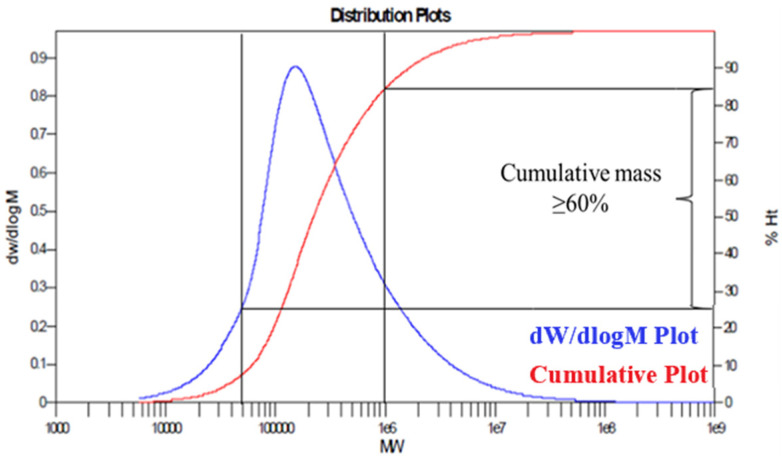
Relative molecular mass distribution image.

The gel permeation chromatography (GPC) test results are shown in Figure 5. 

The GPC test results are shown in Figure 5. The red line represents the cumulative mass distribution, and the blue curve represents the differential distribution of relative molecular mass. It can be observed from the figure that the relative molecular mass of the polymer is distributed between 5 and 100 × 10^4^, the cumulative mass is more than 60%, and the relative molecular mass of 10~30 × 10^4^ is the highest.

The low-molecular-weight polymer is the main product. The gel plugging agent prepared with this polymer has low viscosity and is easy to pump in the wellbore with low pressure on site.

### 2.3. Formulation Optimization of Delayed Crosslinking Low-Molecular-Weight Polyacrylamide Plugging Agent 

#### 2.3.1. Optimization of Polymer Concentration 

**Figure 6:**
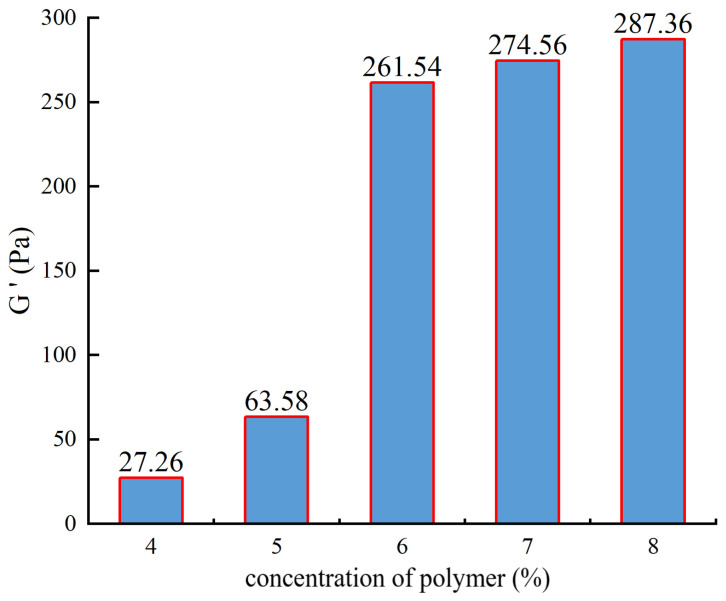
The experimental results of polymer concentration optimization.

When preparing the gel, the polymer concentration was uniformly varied from 4% to 8%, followed by viscoelasticity testing, as shown in Figure 6.

From Figure 6, it can be observed that as the polymer concentration increases, the elastic modulus initially shows a slow increase. However, when the concentration reaches 6%, there is a significant increase in the elastic modulus, followed by a slower growth trend. A higher polymer concentration leads to a greater number of crosslinking points between the polymer macromolecules and the crosslinking agent, resulting in a denser three-dimensional network structure and increased strength. However, when the polymer concentration is too high, many macromolecules do not come into contact with the crosslinking agent and remain unbound, resulting in only a slight increase in strength. Therefore, the optimal polymer concentration selected is 6%.

#### 2.3.2. Concentration Optimization of Crosslinking Agent

**Figure 7:**
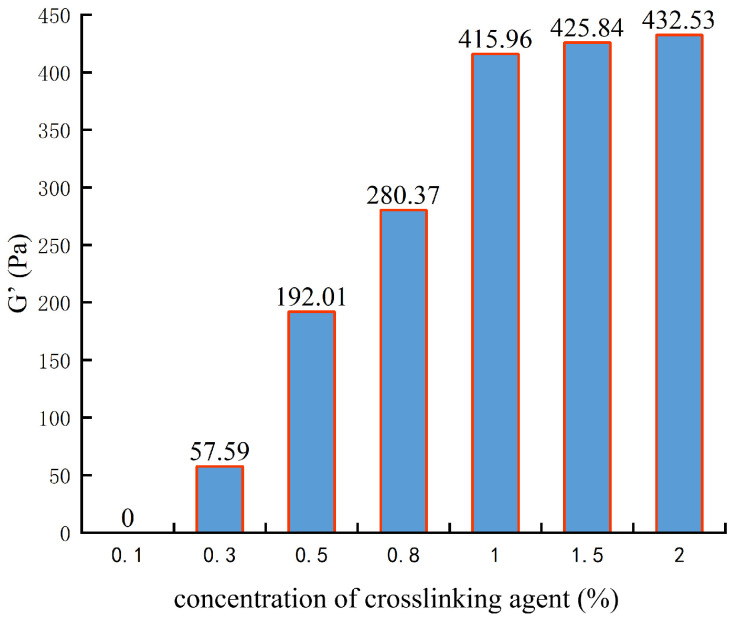
The experimental results of crosslinking agent concentration optimization.

During gel preparation, the crosslinking agent concentration was designed to be 0.1%, 0.3%, 0.5%, 0.8%, 1%, 1.5%, and 2%. Viscoelasticity testing was conducted accordingly, as shown in Figure 7. 

From Figure 7, it can be observed that when the concentration of the crosslinking agent is 0.1%, the polymer does not form a gel structure. When the concentration of the crosslinking agent was 0.1–1%, the gel strength increased slowly, and the increase in the crosslinking agent concentration was beneficial to the improvement in gel strength. When the concentration of the crosslinking agent is greater than 1%, the concentration of the crosslinking agent has little effect on the gel strength, which may be due to the saturation of crosslinking agent and polyacrylamide, so that it is not crosslinked. Therefore, the optimal crosslinking agent concentration is determined to be 1%.

#### 2.3.3. Concentration Optimization of Retarder

**Figure 8:**
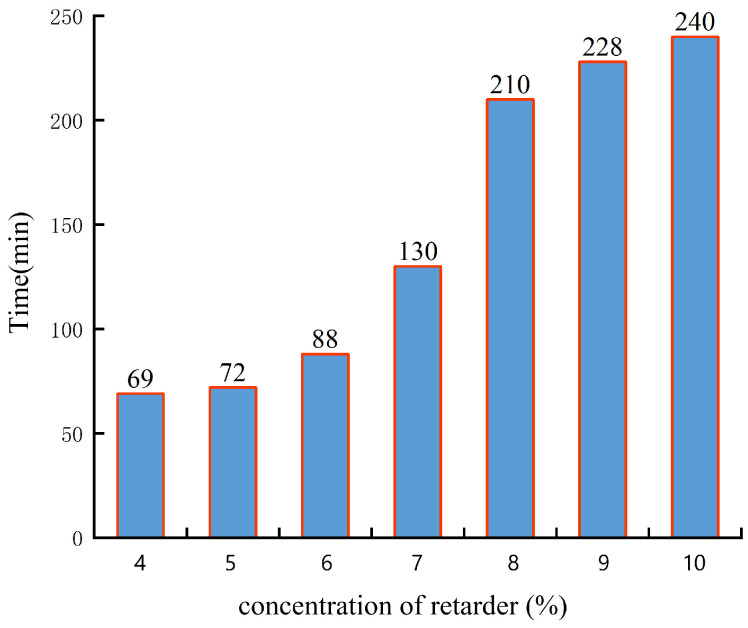
The experimental results of retarder concentration optimization.

After preparing the gel solution, a retardant agent was added at concentrations of 4%, 5%, 6%, 7%, 8%, 9%, and 10%. The prepared solutions were then placed in a high-temperature and high-pressure thickening device. Considering the practical conditions on site and the properties of the polymer itself, the experimental conditions were set at a temperature of 150 °C and a pressure of 60 MPa. The gelation time of the gel was observed, as shown in Figure 8. 

From Figure 8, it can be observed that when the retardant agent concentration is relatively low, the thickening time of the gel remains relatively constant. However, when the concentration reaches 6%, the gelation time rapidly increases. As the concentration reaches 8%, the rate of the increase in gelation time gradually slows down. When the concentration of the retardant agent is further increased, the rate of change in gelation time decreases. The presence of retarder can prolong the gelation time, which is because it can react with the polymer and crosslinking agent to form a slowly hydrolyzed complex intermediate, thereby increasing the crosslinking reaction step and prolonging the gelation time. When the concentration of the retarder is too low, the gelation time is faster, the concentration of the retarder increases, and the gelation time becomes longer. By adjusting the concentration of the retarder, the gelation time can be controlled [28]. Overall, under the conditions of 150 °C, the gelation time of the gel can be controlled by adjusting the concentration of the retardant agent. The time variation is controllable and can be adjusted within the range of 1 h to 4 h.

### 2.4. Comprehensive Properties of Low-Molecular-Weight Polyacrylamide Plugging Agent with Delayed Crosslinking

#### 2.4.1. Rheological Properties

**Figure 9:**
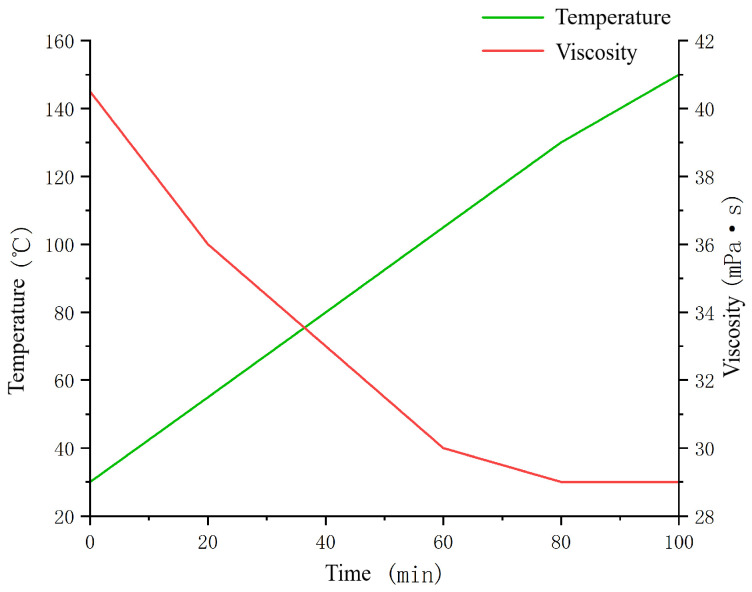
Variable temperature rheological test.

The optimized gel solution formulation consisted of 6% polymer, 1% crosslinking agent, and 8% retardant agent. A 50 mL portion of the gel solution was taken for rheological testing at temperatures ranging from 30 to 150 °C. The results of the testing are depicted in Figure 9.

The results of the temperature-dependent rheological experiment are shown in Figure 9. At room temperature, the viscosity is relatively low, which is advantageous for the transportation of onshore pipelines. As the temperature increases, the viscosity decreases slightly by approximately 15 mPa·s, and the viscosity remains relatively stable.

#### 2.4.2. Temperature Resistance

The prepared gel solution was subjected to high-temperature and high-pressure thickening experiments, with aging temperatures set at 150 °C, 160 °C, and 170 °C, while measuring its viscoelasticity. 

**Figure 10:**
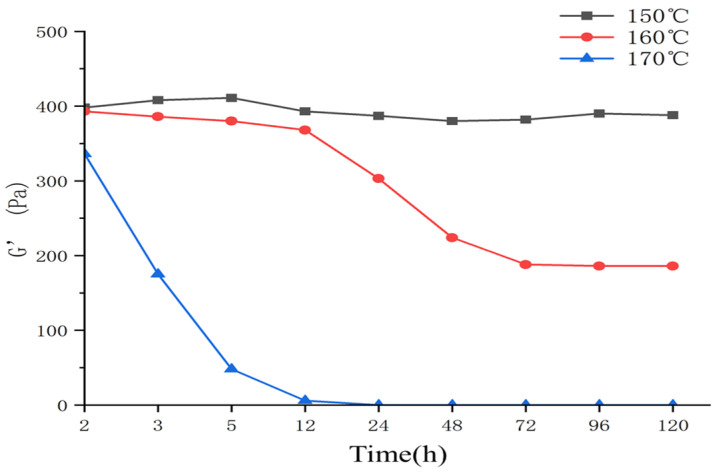
The temperature resistance test.

From Figure 10, it can be observed that the gel strength remains relatively stable at 150 °C. At 160 °C, the gel strength experiences a slight decrease within the first 12 h, but the decrease is not significant. However, after 48 h, the gel strength drops by 40%, indicating that the temperature resistance of the gel sealing material is limited at 160 °C. At 170 °C, the gel strength undergoes a significant decrease within the first 12 h, and afterward, there is virtually no strength remaining. As the temperature increases, the functional groups of the polymer chains undergo changes, resulting in the curling and contraction of the chain ends. The interconnected crosslinking agents and polymers break, leading to a fragile spatial structure. With further temperature increase, the crosslinking bonds continue to break, eventually decomposing into individual polymer chains, which renders the sealing agent ineffective.

#### 2.4.3. Delayed Crosslinking Performance

**Figure 11:**
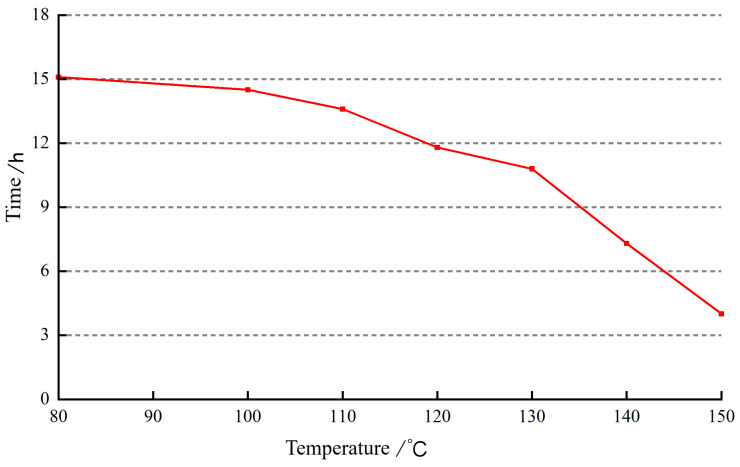
The effect of temperature on crosslinking time.

The prepared gel solution was subjected to high-temperature and high-pressure thickening experiments with aging temperatures set at 80 °C, 100 °C, 110 °C, 120 °C, 130 °C, 140 °C, and 150 °C. The changes in the gelation time of the gel sealing agent at different temperatures were observed, as shown in Figure 11.

As the temperature increases, the crosslinking time of the gel sealing agent gradually decreases. Before 130 °C, the crosslinking time decreases slightly, but after exceeding 130 °C, the crosslinking time starts to decrease significantly. At 150 °C, the crosslinking time is 4 h. As the temperature increases to the critical temperature of the retardant agent, the retardant agent begins to decompose, releasing the crosslinking agent. The higher the temperature, the faster the decomposition of the retardant agent, leading to a faster formation of the sealing gel and poorer delayed crosslinking effect. In this study, the retardant agent used exhibited a significant increase in the decomposition rate at temperatures above 130 °C, resulting in a faster crosslinking speed and a poorer delayed crosslinking effect.

### 2.5. Plugging Performance of Delayed Crosslinking Low-Molecular-Weight Polyacrylamide Plugging Agent

#### 2.5.1. Sealing Characteristics

The evaluation of the sealing efficiency of the polymer gel plugging agent for fractures was conducted using 40–60 (20–40) mesh quartz sand packed into a sand-packed tube. Standard saline water was prepared to simulate formation water, consisting of 2.0% KCl, 5.5% NaCl, 0.45% MgCl_2_, and 0.55% CaCl_2_. The permeability K_1_ of the sand-packed tube was determined using the standard saline water. The gel solution was displaced into the sand-packed tube until a stable flow rate was observed at the outlet. The sand-packed tube was then heated to 80 °C using a heating device and subsequently cooled. After cooling, the permeability K_2_ of the sand-packed tube was measured using standard saline water. The plugging efficiency of the sand-packed tube was calculated according to Equation (1).

(1) $$\eta =\frac{K_{1}-K_{2}}{K_{1}}\times 100\%$$

The results are shown in Table 2.

According to Table 2, it can be observed that under high-temperature conditions, crosslinking occurs, which results in the consolidation of the quartz sand particles and the filling of the quartz sand fractures by the gel plugging agent. This mechanism enhances the sealing efficiency of the gel plugging agent.

#### 2.5.2. Salt Resistance

To prepare a saltwater simulation of formation water, the following composition was used: 25% KCl, 60% NaCl, 6% MgCl_2_, and 9% CaCl_2_. Gel solutions with mineralization levels of 0 mg/L, 1000 mg/L, 5000 mg/L, 10,000 mg/L, 15,000 mg/L, and 20,000 mg/L were prepared using the saltwater. The viscosity of these solutions was measured, followed by high-temperature crosslinking, and finally, the viscoelastic properties were determined:

(1) Influence of mineralization on initial viscosity

**Figure 12:**
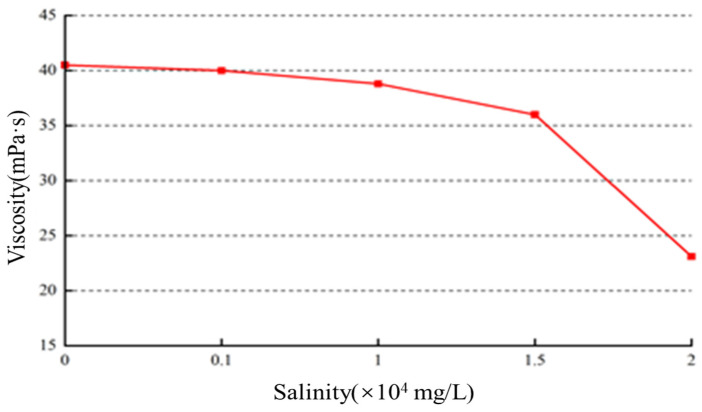
The effect of salinity on initial viscosity.

The prepared gel solutions were subjected to viscosity tests, and the impact of mineralization on the viscosity is shown in Figure 12. 

As the mineralization level increases, the viscosity of the gel solution decreases. When the mineralization level reaches 15,000 mg/L, the viscosity is measured to be 36.1 mPa·s. After that point, the decrease in viscosity becomes more pronounced. The polymer in the gel solution contains a significant number of negatively charged groups. At lower mineralization levels, the electrostatic repulsion between the polymer chains and the surrounding environment causes the polymer chains to be in an extended state. However, when the mineralization level increases to 15,000 mg/L, the metal cations in the environment neutralize the negative charges carried by the polymer, leading to a reduction in electrostatic repulsion.

(2) Influence of mineralization on gelation time

**Figure 13:**
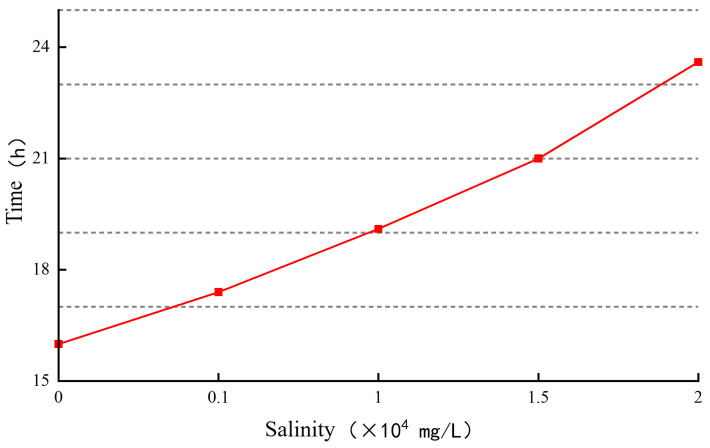
The effect of salinity on gelation time.

High-temperature crosslinking of the gel was performed using a high-temperature and high-pressure thickening instrument with the temperature set at 80 °C. The effect of different mineralization levels on the gelation time is shown in Figure 13. 

As the mineralization level increases, the gelation time gradually increases. When the mineralization level reaches 15,000 mg/L, the gelation time reaches 21 h. As the mineralization level continues to increase, the rate at which the gelation time increases slows down.

The increase in salt concentration enhances the salt sensitivity, causing the compression of the double layer and the severe contraction and curling of the polymer chains. This leads to a decrease in the number of crosslinking sites between the crosslinking agent and the polymer, resulting in a slower formation of the network structure and an increase in gelation time. When the salt concentration reaches a certain value, the compression of the double-layer polymer reaches its limit, and the decrease in crosslinking points becomes minimal. As a result, the gelation time essentially stops increasing.

(3) Influence of salinity on gel strength

**Figure 14:**
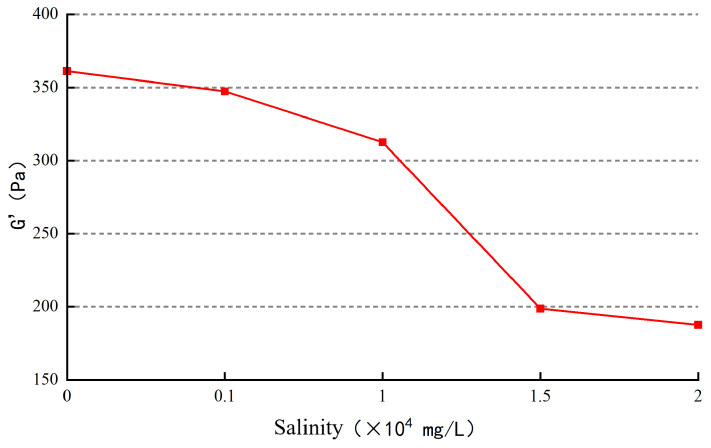
The effect of salinity on gel strength.

The gel was subjected to viscoelastic testing, and the variation in gel strength with mineralization is shown in Figure 14. 

As the mineralization level increases, the gel strength exhibits a decreasing trend. When the mineralization level reaches 15,000 mg/L, the gel strength levels off, with an elastic modulus of approximately 190 mPa·s. The increase in salt concentration leads to the compression of the double layer, causing the polymer chains to curl and reducing the number of crosslinking points. This results in a well-developed grid structure in the planar direction of the gel but fewer three-dimensional structures, leading to a decrease in gel strength. As the salt concentration continues to increase, the number of crosslinking points reaches its lowest value, and the grid structure stabilizes. Consequently, the gel strength remains relatively unchanged.

#### 2.5.3. Pressure Test

To test the sealing capability of the plugging agent under pressure, rock samples with different fracture widths were prepared. The gel was fully crosslinked within the fractures under conditions of 80 °C. The drilling fluid was water with 6% bentonite. The drilling fluid was then applied to the fractures to increase the pressure, and the amount of leakage at the outlet end within 1 min was recorded. Based on the experimental results, the pressure-bearing capacity of the gel plugging agent under different fracture widths was determined, as shown in Table 3. When the fracture width is less than 0.1 mm, the pressure-bearing capacity can exceed 9 MPa. When the fracture width is less than 2 mm, the pressure-bearing capacity ranges from 6 to 7 MPa.

After gelation, there is a viscous force between the gel plugging agent and the contact surface of the fracture. When the applied pressure is lower than the viscous force, the gel plugging agent can continue to seal the fracture. The greater the pressure applied by the drilling fluid on the gel, the poorer the sealing effect of the gel, and the lower the pressure-bearing capacity.

## 3. Conclusions

The conclusions of this study are as follows:

(1) The low-molecular-weight polyacrylamide prepared using hydrogen peroxide has a controllable gelation time and good flowability before crosslinking. This is more advantageous for pumping during on-site construction. Additionally, the low-molecular-weight polyacrylamide prepared using hydrogen peroxide has higher strength after high-temperature crosslinking.
(2) A low-molecular-weight polyacrylamide was developed, and the optimal synthesis conditions were determined. The optimal conditions include a monomer concentration of 9%, an initiator concentration of 3.5%, and a synthesis temperature of 65 °C.
(3) Using the newly developed low-molecular-weight polyacrylamide as a key treatment agent, a delayed crosslinking formula for the low-molecular-weight polyacrylamide plugging system was optimized. The specific formula consists of 6% polymer, 1% crosslinking agent, and 8% retarder.
(4) The newly developed delayed crosslinking low-molecular-weight polyacrylamide plugging agent has stable rheological properties, and the temperature resistance can reach 150 °C. By adjusting the concentration of the retarder, the gelation time can be controlled, and the maximum can be up to 4 h at 150 °C.
(5) The maximum salt tolerance of the delayed crosslinking low-molecular-weight polyacrylamide plugging agent is 15,000 mg/L, and the plugging rate of the gel plugging agent is more than 95%.

## 4. Materials and Methods

### 4.1. Synthetic Materials and Instruments

The synthetic materials used in this study include acrylamide monomer (AM) as the monomer; hydrogen peroxide and ammonium persulfate as initiators; chromic acetate as the crosslinking agent; and sodium lactate as the retarder.

Acrylamide (AM), chemically pure grade, was obtained from China National Pharmaceutical Group Chemical Reagent Co., Ltd(Chengdu, China). In addition, 30% hydrogen peroxide, chemically pure grade, was obtained from China Millipore Reagent. Ammonium persulfate, electrophoresis grade, was obtained from China Aladdin Reagent. Alkaline chromium acetate, chemically pure grade, was obtained from Aladdin Reagent. Sodium lactate, chemically pure grade, was obtained from China Aladdin Reagent.

The synthetic apparatus consisted of the following equipment: a graduated cylinder with a volume of 250 mL, a beaker with a volume of 250 mL, a constant-pressure dropping funnel with a volume of 50 mL, a three-necked flask with a volume of 250 mL, a coiled condenser with a length of 300 mm, a magnetic stirring constant-temperature water bath, a nitrogen cylinder, and a stirrer.

### 4.2. Synthesis of Polymer

**Figure 15:**
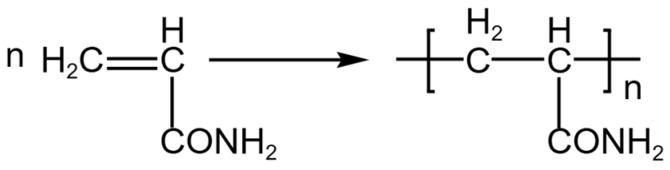
Synthesis of Polymer.

A predetermined amount of initiator and acrylamide (AM) was weighed and mixed with a solvent. The mixture was then separately poured into the constant-pressure dropping funnel and the three-necked flask for later use. The three-necked flask and the constant-pressure dropping funnel were assembled and secured in a water bath. The stirrer was turned on, and nitrogen gas was introduced for a period of 10–15 min. Afterward, the coiled condenser was opened, and the magnetic stirring constant-temperature water bath was heated to the predetermined temperature. Within the time frame of 10–15 min, the initiator was added dropwise. After stirring for 3 h, polyacrylamide was obtained. The synthesis principle of polymer is illustrated in Figure 15.

### 4.3. Preparation of Gel Solution

In total, 50 mL of water was added to a beaker, and 6.4% polymer, 1.6% crosslinking agent, and 5% acrylamide were added and mixed well under the stirring of the magnetic rotor.

### 4.4. Material Characterization

In this paper, the performance evaluation of the synthesized polymer was carried out by referring to the evaluation method of polymer gel for oil recovery ‘SYT 6296-1997 determination of polymer gel strength for oil recovery-rheological parameter method’.

(1) Rheological testing: The viscosity of the gel solution before crosslinking was measured using a HAKKE MARS III rheometer. The shear rate was set at 170 s^−1^, and the shear time was 20 min. The viscosity of the gel solution before crosslinking reflects its rheological properties during the injection process into the formation. A lower viscosity corresponds to a lower injection pressure and a lower probability of retention in the wellbore.
(2) High-temperature crosslinking: The gel solution was prepared at room temperature, and its viscosity was measured. The gel solution was then placed in a high-temperature reaction vessel and heated to a predetermined temperature for a specific duration of high-temperature crosslinking. After gelation, the sample was removed and cooled. The crosslinking status of the gel can be visually observed during this process, allowing for evaluation of the gel’s crosslinking state.
(3) Viscoelasticity testing: After cooling the high-temperature crosslinked gel to room temperature, 5 mL of gel was taken out, and the viscoelasticity test was carried out with a HAKKE MARS III rheometer. The test conditions were temperature 30 °C, fixed scanning frequency 1 Hz, and stress 10 Pa. The average value of elastic modulus G’ was obtained. For polymer gels, a higher elastic modulus indicates greater strength and better crack sealing performance.
(4) Gel permeation chromatography: The number-average molecular weight (M_n_) and weight-average molecular weight (M_w_) of the synthesized polymer samples were measured with gel permeation chromatography (GPC, from Beijing, China, SHIMADZU Co., DGU-20A3R), and the polydispersity index (PDI) was obtained by dividing M_w_ by M_n_. Polyethylene glycol (PEG) was used as eluent with 0.1 mol/L NaNO_3_ solution.

## Figures and Tables

**Table 1:** Flow description and elastic modulus of different initiators.

Initiator	Character	G’/Pa	Viscosity-Average Molecular Weight
3% H_2_O_2_	Free flowing	205	173,100
3.5% H_2_O_2_	Free flowing	300	569,800
5% H_2_O_2_	Free flowing	236	976,500
3.5% (NH_4_)_2_S_2_O_8_	Mobility difficulties	261	1,566,000
7% (NH_4_)_2_S_2_O_8_	Mobility difficulties	229	2,384,200

**Table 2:** Experimental results of plugging performance.

Mesh	Permeability before Plugging K_1_/mD	Permeability after Plugging K_2_/mD	η/%
40~60	9.1	0.241	97.35
20~40	17.1	0.512	97.01

**Table 3:** Pressure-bearing capacity experimental results.

Pressure /MPa	Filtration Loss/mL					
	0.05 mm	0.1 mm	0.2 mm	0.5 mm	1 mm	2 mm
0.5	0	0	0	0	0	0
5	0	0	0	0	0	0
6	0	0	0	0	0.255	0.95
7	0	0	0.041	0.108	0.587	2.822
8	0	0	0.043	0.653	3.804	6.05
9	0	0	0.046	1.305	9.655	8.766
10	0	0.13	0.512	2.428		

## Data Availability

The raw/processed data required to reproduce these findings cannot be shared at this time as the data also form part of an ongoing study.
